# Effect of Dietary Fiber Supplementation on Metabolic Endotoxemia: A Protocol for Systematic Review and Meta-Analysis of Randomized Clinical Trials

**DOI:** 10.3390/mps6050084

**Published:** 2023-09-11

**Authors:** Yazan Ranneh, Abdulmannan Fadel, Abdah Md Akim, Iskandar Idris, Bolaji Lilian Ilesanmi-Oyelere, Leila Cheikh Ismail

**Affiliations:** 1 Department of Nutrition and Dietetics, College of Pharmacy, Al Ain University, Abu Dhabi P.O. Box 112612, United Arab Emirates; 2 Research Institute for Sport and Exercise Sciences, School of Sport and Exercise Sciences, Liverpool John Moores University, Liverpool L3 3AF, UK; a.fadel@ljmu.ac.uk; 3 Department of Biomedical Sciences, Faculty of Medicine and Health Sciences, Universiti Putra Malaysia, Serdang 43400, Malaysia; abdah@upm.edu.my; 4 School of Medicine, University of Nottingham, Royal Derby Hospital, Derby DE22 3NE, UK; iskandar.idris@nottingham.ac.uk; 5 College of Health, Massey University, Palmerston North 4442, New Zealand; b.ilesanmi-oyelere@massey.ac.nz; 6 Department of Clinical Nutrition and Dietetics, University of Sharjah, Sharjah P.O. Box 72772, United Arab Emirates; lcheikhismail@sharjah.ac.ae; 7 Nuffield Department of Women’s & Reproductive Health, University of Oxford, Oxford OX3 9DU, UK

**Keywords:** metabolic endotoxemia, dietary fiber, meta-analysis, systematic review, low-grade inflammation, clinical trials

## Abstract

**Introduction:** Metabolic endotoxemia (ME) is the main cause of sub-clinical chronic inflammation, which subsequently triggers the onset of several chronic diseases. However, recent reports have indicated that dietary fiber (DF) contributes significantly to ameliorating ME and inflammation. This protocol aims to provide an outline of all procedures in synthesizing the available data on the effect of DF against ME. **Methods:** Following the PRISMA 2020 guidelines for preparing protocols, this protocol was registered in the International Prospective Registry of Systematic Reviews (PROSPERO) with registration number (CRD42023417833). In this review, we specifically focused on the inclusion of clinical trials that met the following criteria: they were published or available as preprints, employed random, quasi-random, or cross-over designs, and were exclusively documented in the English language. Clinical medical subject headings (MeSH) as search terms were used on prominent databases such as MEDLINE, COCHRANE library, PubMed, World Health Organization International Clinical Trials Registry Platforms, and US National Institutes of Health Ongoing Trials Register Clinicaltrials.gov. **Results and discussion:** This protocol will guide the exploration of articles that report changes in ME biomarkers in subjects supplemented with DF. The findings of this protocol will ensure a comprehensive evaluation of available evidence, provide a quantitative summary, identify patterns and trends, enhance statistical power, and address heterogeneity, which collectively will clarify the optimal types, doses, and duration of DF interventions for managing ME and low-grade inflammation. **Ethics and dissemination:** The quantitative data of clinical trials will be collected, and a meta-analysis will be performed using RevMan V.5.3 software. Therefore, no ethical approval is required.

## 1. Introduction

Metabolic endotoxemia (ME) occurs when a low-grade of lipopolysaccharide (LPS) produced by Gram negative bacteria enters the bloodstream due to diet-induced changes in the gut microbiome and/or in the intestinal permeability [1]. Upon entering the circulatory system, LPS associates with LPS binding protein (LBP), which in turn binds to cluster differentiation 14 (CD14). This complex then interacts with membrane-bound CD14 and the myeloid differentiation factor 2/Toll-like receptor-4 (TLR4) receptor complex expressed on cells, subsequently leading to enhanced activation of the NF-κB pathway and secretion of pro-inflammatory cytokines, such as TNF-α and IL-6 [2]. The continuous secretion of pro-inflammatory cytokines initiates a state of sub-clinical chronic inflammation [3]. This pathophysiological phenomenon has been implicated in the development and progression of various chronic diseases, including diabetes type 2, atherosclerosis, non-alcoholic fatty liver (NAFLD), obesity, and chronic kidney diseases [4]. 

Numerous studies have investigated the immediate effects of high-calorie, high-fat, and high-carbohydrate meals on postprandial endotoxemia and subsequent physiological sequelae. In healthy individuals, consumption of a single high-fat meal containing 900 calories—consisting of 50 g of butter on three slices of toast—resulted in a significant 50% increase in median plasma endotoxin levels from baseline, as well as postprandial inflammation [5]. A population-based study comprising 1015 healthy male subjects was conducted to explore the relationship between energy intake and the plasma levels of LPS. The results of the study demonstrated a significant correlation between the two variables. Furthermore, an analogous association was observed in mice that were exposed to high-fat or high-energy, high-carbohydrate diets for a period of four weeks [1,6,7]. In mice, the consumption of a high-fat diet has been found to lead to an increase in the permeability of the intestines. This increase is caused by the inhibition of mRNA expression of tight junction-related factors, namely zonula occludens-1 (ZO-1) and occludin, in the cells that line the intestinal walls [1]. Stimulation of TLR-4 by elevated levels of intestinal LPS has been shown to elicit the disruption of tight junctions in intestinal epithelial cells, leading to compromised integrity of the intestinal barrier [1].

The amelioration and/or treatment of ME is of paramount importance due to its strong association with a wide range of chronic diseases. The current body of empirical evidence indicates that nutritional interventions that target the reduction of circulating endotoxin levels may have significant health implications for the human population [8]. Several studies have demonstrated that dietary modifications can positively modulate the gut microbiota composition, leading to improved gut barrier function and reduced endotoxin translocation from the gut into circulation. Such interventions include the incorporation of prebiotics, probiotics, and dietary fibers (DF) in the diet, as well as the adoption of a Mediterranean or plant-based diet. In a recent study, a dietary intervention designed to target gut microbiota for the management of chronic low-grade inflammation and metabolic syndrome demonstrated notable improvements in gut ecology, intestinal permeability, plasma endotoxin activity, inflammatory markers, and metabolic health, including weight, insulin sensitivity, lipid profiles, and blood pressure. These positive outcomes were observed within a 9-week period among obese patients who underwent the intervention [9]. 

### 1.1. Description of the Intervention

DF is a complex component of food that comprises carbohydrate polymers and oligomers that cannot be digested in the small bowel; hence, it moves to the large bowel while retaining its chemical structure [10]. The physiochemical characteristics of DF, such as solubility, viscosity, and fermentability, determine its functionality in the gut and accessibility to gut microbes. Gut microbiota can ferment most soluble fibers partially or completely, depending on their chemical structure. Various definitions of DF exist, based on their chemical compounds, functional compounds, or both, owing to the wide range of non-digestible fibers found in nature. The European Food Safety Authority (EFSA) defines DF as “non-digestible carbohydrates plus lignin”, which includes non-starch polysaccharides (NSP) such as cellulose, hemicelluloses, and pectins, hydrocolloids such as gums, mucilages, β-glucans, resistant oligosaccharides, resistant starch, and lignin, which are associated with DF polysaccharides [11]. 

Prebiotics represent a specific subset of DF that exhibit selective fermentability by gut microbiota, resulting in improved host health [12]. Although DF is often equated with prebiotics, not all fibers possess prebiotic properties because their in vivo fermentability varies significantly among individuals [13]. The term “prebiotic” was first defined more than a quarter century ago by Gibson and Roberfroid as a non-digestible food ingredient that selectively stimulates the growth and/or activity of a limited number of bacteria in the colon, thereby providing health benefits to the host. This definition has since been updated to encompass a broader range of substrates that are selectively utilized by host microorganisms, conferring health benefits [14]. The previous definition has also been confirmed by The International Association for Probiotics and Prebiotics (ISAPP) and allows for the inclusion of non-fiber substrates in the prebiotic classification [15,16]. Recently, the immune health benefits of inulin, fructo-oligosaccharides (FOS), galacto-oligosaccharides (GOS), and xylooligosaccharides (XOS) have been extensively studied in modulating the response of inflammation [17]. 

A cross-sectional study has found a negative correlation between DF consumption and LPS binding protein [18]. In addition, a cross-over study was undertaken to investigate the impact of DF on the levels of endotoxins. The fiber-rich meal was comprised of oatmeal, milk, raisins, peanut butter muffins, and orange juice, with a macronutrient composition of 58% carbohydrates, 27% fats, and 15% proteins. The findings of the study suggest that the consumption of a fiber-enriched breakfast may mitigate the rise in endotoxin levels in the blood, as compared to a standard breakfast [19]. In rigorous intervention trials, oligofructose and inulin were examined on subjects with obesity, overweight, or type 2 diabetes at dosages ranging from 10 to 21 g for a duration of 8 to 12 weeks [20,21,22]. Intriguingly, the outcomes of two out of the three conducted studies provided compelling evidence, indicating a statistically significant decline in circulating LPS levels among the participant cohorts [20,21]. Moreover, the administration of galacto-oligosaccharide, a soluble prebiotic DF, has reduced LPS levels and suppressed appetite among overweight adults [23]. 

### 1.2. How the Intervention Might Work

LPS, chemically resembling a glycolipid, is absorbed in a manner similar to other dietary lipids during the process of intestinal lipid uptake. This involves the enzymatic breakdown of dietary lipids by lipase enzymes, primarily pancreatic lipase, with colipase as its co-factor. The resulting lipids are reassembled into chylomicrons, large particles that are released into the lymphatic system for distribution throughout the body. The presence of LPS within chylomicrons suggests a trans-cellular uptake mechanism for LPS [24]. Apart from being taken up through chylomicrons, LPS can also directly enter intestinal cells and can be released into the bloodstream. This process of trans-cellular uptake is rapid and occurs within a span of 30 min. It relies on the presence of long-chain fatty acids and bile salts in the intestinal lumen, thereby being associated with fat consumption. Unlike the LPS carried by chylomicrons, the LPS taken up through this molecular pathway is readily capable of initiating an inflammatory response [25]. 

DF exhibits a plethora of mechanisms that mitigate the effects of ME. One such mechanism involves impeding the absorption of chylomicrons, which may carry LPS. Notably, pectins have demonstrated the ability to interact with bile acids and phospholipids, resulting in a reduction of surface-active components and decreased solubility of dietary fats [26]. Furthermore, compelling evidence suggests that DF possesses the capacity to bind with LPS within the intestinal lumen, effectively suppressing the translocation of LPS into the bloodstream. By regulating the functional movement of both the small and large intestines, DF diminishes the exposure of intestinal epithelial cells to harmful substances, including LPS [27]. Distinct types of DF act as TLR ligands, instigating downstream phosphorylation of IκB and influencing cytokine production. Specifically, two variants of resistant starch (RS), namely RS type 2 and type 3, predominantly bind to TLR2 and TLR5, respectively [28]. Moreover, chitosan oligosaccharide has exhibited superior inhibitory activity against both Gram-positive and Gram-negative bacteria, such as *Bacillus cereus*, *E. coli*, *Yersinia enterocolitica*, and *Bacillus licheniformis*, when compared to natural chitosan [29]. DF-derived microbial metabolites, including short-chain fatty acids (SCFAs), play a pivotal role in promoting intestinal immune homeostasis by inhibiting histone deacetylases (HDACs) and engaging various receptor-mediated pathways, such as GPCR41 and peroxisome proliferator-activated receptors (PPARs) [27].

### 1.3. Why It Is Important to Do This Review 

The investigation of changes in the serum or plasma levels of LPS among individuals afflicted with ME remains a realm where limited scientific attention has been devoted. However, discerning such alterations holds immense promise as an enticing therapeutic avenue, with the potential to mitigate the burden of chronic diseases by effectively mitigating low-grade inflammation. In a 2019 randomized controlled trial investigating the impact of a high-fiber diet on ME in obese individuals, a 4-week intervention revealed decreased plasma endotoxin levels and enhanced markers of metabolic health, including improved insulin sensitivity and lipid profiles [30]. Another randomized controlled trial investigated the effects of DF supplementation on markers of inflammation and metabolic health in overweight and obese individuals. The study found that supplementation with soluble fiber (inulin) for 12 weeks led to reduced levels of endotoxin in the blood and improvements in inflammation markers, such as C-reactive protein (CRP) [31]. 

After conducting a comprehensive search of systematic reviews pertaining to our chosen topic, we have identified and obtained access to one relevant systematic review. This study was not specific for humans and did not provide a quantitative summary of the collective results [32]. Although these studies provided a highlight of using DF against ME, it was clearly noted that more robust clinical trials are needed to establish definitive conclusions and direct recommendations on the use of DF on ME. Additionally, individual variations in response to dietary interventions and the specific types and amounts of fiber consumed may influence the outcomes. Thus, conducting a systematic review and meta-analysis on the effect of DF against ME ensures a comprehensive evaluation of available evidence, provides a quantitative summary, identifies patterns and trends, enhances statistical power, and addresses heterogeneity, which collectively will clarify the optimal types, doses, and duration of fiber interventions for ME and low-grade inflammation. 

### 1.4. Objective

To systematically review, assess, summarize, and interpret clinical trials studies on how DF intervention could reduce serum or plasma LPS concentrations in patients with ME. In addition, this study will critically summarize how DF administration affects inflammatory cytokines in patients with ME and low-grade inflammation.

## 2. Methods

The protocol of the current systematic review is registered in the International Prospective Registry of Systematic Reviews (PROSPERO) with registration number (CRD42023417833).

### 2.1. Criteria for Considering Studies for This Review

#### 2.1.1. Type of Studies

In this review, we specifically focused on the inclusion of clinical trials that met the following criteria: they were published or available as preprints, employed random, quasi-random, or cross-over designs, and were exclusively documented in the English language. 

#### 2.1.2. Types of Participants

Subjects who fulfill the diagnostic criteria of ME and are treated with DF will be included. Based on the literature, the diagnostic criteria of ME is elevated levels of LPS along with inflammatory cytokines [1]. No limitations will be imposed on the inclusion of participants based on the following factors: age, gender, genotype, antibiotic use, and LPS concentration. 

#### 2.1.3. Types of Intervention

Eligible interventions include the intake of DF. The DF should be specified clearly and administrated for a minimum of two weeks. Clinical trials that administrated DF with co-intervention are eligible if the co-intervention is present in both groups, the treatment and control. In vitro trials or trials that studied the effect of probiotics or synbiotics will be excluded. 

#### 2.1.4. Comparators/Control

The control group will consist of individuals who were administered either placebo or non-pharmacological treatments that do not contain DF for a minimum of two weeks. A baseline comparison of patients before the intervention is also included. 

## 3. Outcomes

### 3.1. Types of Outcome Measure

The primary outcome of this review will be the measurement of LPS or LBP concentrations in blood or feces using LAL assay or ELISA method in response to DF intervention. The secondary outcomes are serum or plasma concentrations of TNF-α, IL-8, IL-6, IL-1β, C-reactive protein, NF-κB, CD-14, IFN-γ, adipocytokines, occludins, claudin 5, zonula occludens 1, HBA1c, and lipid profile.

### 3.2. Search Methods for Identification of Studies

We will conduct a comprehensive search for published trials, without imposing any restrictions on year of publication, or publication status or language.

#### 3.2.1. Electronic Search 

One of our information specialists will search in the following databases, using Employing a blend of textual expressions and medical subject heading (MeSH) indexing terms as outlined in supplementary (Table S1).

- Cochrane Central Register of Controlled Trial (CENTRAL, current issues) in the Cochrane Library;
- MEDLINE Ovid SP;
- PubMed;
- World Health Organization International Clinical Trials Registry Platforms;
- US National Institutes of Health Ongoing Trials Register Clinicaltrials.gov.

We will refine the search outcomes by implementing filters for clinical trials, following the guidelines set forth in the *Cochrane Handbook for Systematic Reviews of Interventions*, thus ensuring a focused exploration within the realm of clinical research [33]. 

#### 3.2.2. Searching Other Resources 

We will scrutinize the bibliographies of the included trials and any relevant systematic reviews found to identify additional references pertaining to relevant trials. Additionally, we will proactively engage with domain experts and relevant organizations to acquire supplementary information regarding trials of importance. We will employ a multifaceted approach to identify unpublished trials, encompassing proactive communication with domain experts in the respective field. To ensure comprehensive coverage, we will meticulously scan the abstracts presented at major international congresses during the three years preceding our search. This strategy aims to capture any studies that have been presented at these events but have yet to be fully published. Additionally, we recognize the significance of linguistic diversity and will diligently pursue the acquisition of translations for papers that necessitate such measures. 

### 3.3. Data Collection and Analysis

Our data collection and analysis will be conducted in accordance with the methodologies prescribed in the *Cochrane Handbook for Systematic Reviews of Interventions* [33]. These established methods serve as the foundation for our rigorous and systematic approach, ensuring the reliability and validity of our findings.

### 3.4. Selection of Studies

**Figure 1:**
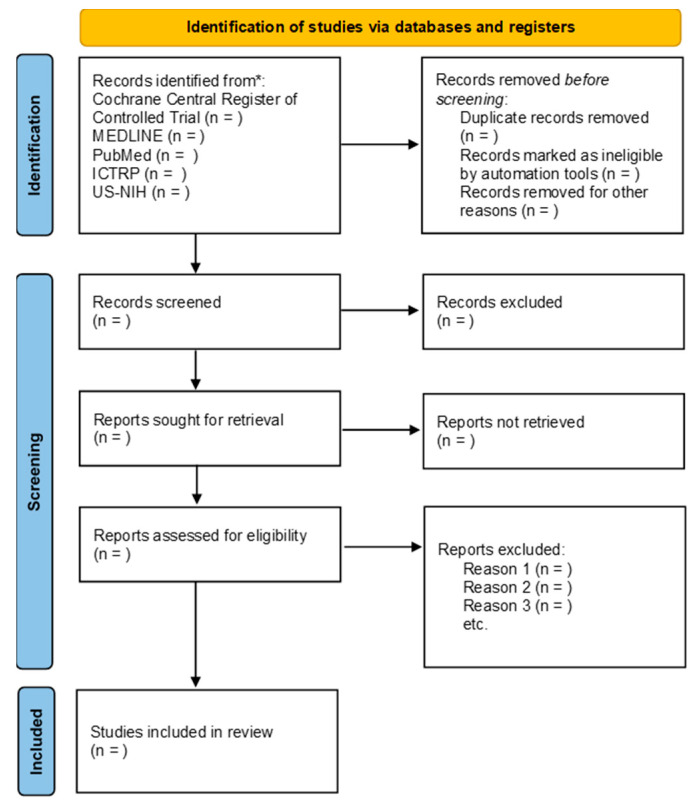
PRISMA 2020 flow diagram for the process of systematic review and meta-analysis.

Two diligent review authors will independently examine the titles and abstracts derived from the literature search, utilizing the robust platform of Covidence (www.covidence.org, accessed on 17 July 2023). Studies that evidently fail to meet the inclusion criteria will be promptly discarded. For studies that exhibit potential alignment with our inclusion criteria or lack adequate information to make a conclusive determination, we will obtain the full reports for further analysis. The review authors will independently evaluate these reports to ascertain whether they meet the inclusion criteria. In the event of any disagreements, resolution will be sought through discussion and, if necessary, consultation with a third review author. The studies rejected at any stage, and the primary reason for their exclusion will be documented in the tables outlining the characteristics of excluded studies. A comprehensive and transparent depiction of the selection process will be recorded to create a PRISMA flow diagram [34] (Figure 1). 

### 3.5. Data Extraction and Management

Two independent review authors will carry out the meticulous task of data extraction utilizing specially designed data extraction forms. To ensure the effectiveness and reliability of the forms, a pilot testing phase will be conducted using two selected studies. In the event of any disagreements or discrepancies, a third author will be consulted for resolution and consensus. The extraction of relevant data will be performed from full-text articles that meet the established inclusion criteria. This comprehensive data collection process will encompass extracting and documenting relevant information such as:

- Participant characteristics (e.g., age, gender, genotype, phenotype, pancreatic status);
- trial characteristics and design (e.g., RCTs or quasi-RCT);
- interventions and comparator (e.g., type of fiber-prebiotic, dose, duration);
- outcome data—reported separately for each outcome.

In cases where a single trial reports multiple trial arms, we will selectively include only the arms that are relevant to our study. To ensure accuracy and reliability, one review author will manually input the extracted data into Review Manager (RevMan 2014), while another author will carefully double-check the entered data for consistency and correctness. In situations where the information or data is ambiguous or incomplete, we will proactively reach out to the authors of the respective studies to seek clarification and obtain additional details as needed.

### 3.6. Assessment Risk of Bias in the Included Studies 

The risk of bias in the included studies will be assessed following the guidelines of the Cochrane Collaboration, utilizing the ROB (Risk of Bias) tool (Higgins and Altman, *Cochrane Handbook for Systematic Reviews of Interventions: Cochrane Book* Series, 2008; 187–241). This tool will evaluate various domains, including random sequence generation, allocation concealment, blinding of participants and outcome assessors, incomplete outcome data, selective reporting, and other potential sources of bias. The risk of bias 2.0 tool will be used for risk of bias assessment in the included studies, utilizing RevMan V.5.3 software. Two independent reviewers will conduct the assessments, and any conflicts will be resolved through discussion. If needed, a third opinion will be sought from another reviewer to ensure robust and reliable assessments.

For each domain, we will assess the risk of bias in the included studies and assign them to one of three categories: low, high, or unclear risk of bias. This assessment will be carried out in accordance with the established guidelines for evaluating the risk of bias as outlined in the *Cochrane Handbook for Systematic Reviews of Interventions* [33]. By applying this standardized framework, we aim to provide an objective evaluation of the quality and potential bias within each study.

### 3.7. Measures of Treatment Effect

In analyzing dichotomous outcomes, we will quantify the treatment effect using risk ratios (RR) accompanied by corresponding 95% confidence intervals (CIs). For continuous outcomes, if the studies employ the same scales and methodologies, we will express the treatment effect as the mean difference (MD) with a 95% CI. However, if the studies assess the same continuous outcome using different methods, we will estimate the treatment effect using the standardized mean difference (SMD) with 95% CIs. The presentation of SMDs will be in standard deviation (SD) units. To interpret the magnitude of the treatment effect, we will rely on the following thresholds: an SMD of 0.2 indicates a small effect, 0.5 represents a moderate effect, and 0.8 signifies a large effect, following the guidelines outlined in Section 12.6.2 of the *Cochrane Handbook for Systematic Reviews of Interventions*.

### 3.8. Unit of Analysis Issue

The primary unit of analysis in our study will be the individual participants. In cases where studies involve more than two intervention groups, we will conduct multiple pairwise comparisons between all possible pairs of intervention groups. To prevent duplication, any shared intervention groups will be evenly distributed among the comparisons. For dichotomous outcomes, both the number of events and the total number of participants will be divided accordingly. For continuous outcomes, only the total number of participants will be divided, while the means and standard deviations will remain unchanged.

Cross-over studies will be considered if the data are reported separately before and after the cross-over period. However, we will only utilize data from the initial phase for our analysis. In the event that cluster-randomized controlled trials (cluster-RCTs) are identified, we will include study data if the authors have appropriately accounted for the clustering effect using suitable statistical methods.

### 3.9. Dealing with Missing Data

In cases where data are missing or studies have not provided sufficient detail, we will make efforts to contact the authors to request the necessary information. If studies report standard errors without reporting standard deviations, we will attempt to estimate the missing standard deviations using appropriate statistical tools and calculators [33]. Studies that fail to report measures of variance will be deemed at high risk of selective reporting bias.

### 3.10. Assessment of Heterogeneity

To assess the level of heterogeneity among the trials included in each analysis, we employed the I^2^ statistic. In cases where substantial heterogeneity was identified, indicated by a value greater than 50%, we reported this finding and conducted pre-specified subgroup analyses to explore potential causes for the observed heterogeneity. This systematic approach allowed us to investigate and analyze any significant variations or differences among the trials, providing valuable insights into the underlying factors contributing to the heterogeneity of the results.

### 3.11. Assessment of Reporting Biases

In order to mitigate the potential for reporting bias arising from trial non-publication or selective outcome reporting, we will employ a comprehensive search strategy, incorporating diverse sources such as trial registries. This inclusive approach is designed to minimize the risk of overlooking relevant trials and outcomes. To further address the issue of bias, we will utilize funnel plots, a statistical technique recommended by Sterne (2011), when a substantial number of trials (at least 10 trials) have reported a specific outcome. The implementation of funnel plots will enable us to evaluate the presence of bias by visually examining the distribution of study results. Through this analytical tool, we aim to quantitatively and qualitatively assess the potential impact of reporting bias, enhancing the robustness and reliability of our findings.

### 3.12. Data Synthesis

If the initial screening of relevant randomized controlled trials (RCTs) has revealed that the outcomes reported in the included studies are not homogeneous, the meta-analysis will not be performed. Therefore, a qualitative analysis will be performed to synthesize the findings of the included studies, along with a critical appraisal of the outcomes reported. However, if we discovered after the data extraction process that certain outcomes are homogeneous across some of the studies, a meta-analysis of those specific outcomes may be conducted.

### 3.13. Subgroup Analysis and Investigation of Heterogeneity

In the presence of heterogeneity, we will diligently explore potential underlying factors contributing to this variation and employ methodologies described in the *Cochrane Handbook for Systematic Reviews of Interventions* to address them. However, we will not perform a comprehensive meta-analysis if substantial heterogeneity persists despite these efforts, without any plausible explanations or resolutions. 

In the event of having a sufficient number of studies (at least 10 studies), we will conduct subgroup analyses to explore potential effect modifiers. Subgroup analyses will be performed based on various factors, including age, sex, or gender (if primary studies provide separate data for these factors), ME activity, study duration (long-term: ≥4 weeks or short-term: <4 weeks), type of fiber, type of intervention (fiber supplementation, high-fiber foods), and dosage. By undertaking these subgroup analyses, we aim to gain insights into potential variations in treatment effects across different subpopulations and contextual factors, thereby enhancing the comprehensiveness and applicability of our findings. 

### 3.14. Sensitivity Analysis

If we identify a sufficient number of studies to include in a meta-analysis, we will conduct a sensitivity analysis to evaluate the robustness of our findings. This sensitivity analysis will involve carefully considering the inclusion or exclusion of trials that we have determined to have a high or unclear risk of selection bias, following the rigorous criteria outlined in our assessment of the risk of bias in the included studies. Additionally, we will perform an additional sensitivity analysis that explores the effects of including or excluding trials with a high risk in the domains of performance bias and detection bias.

## 4. Discussion

To the best of our knowledge, this systematic review and meta-analysis marks the first comprehensive investigation into the effects of DF supplementation on ME. The interaction between dietary interventions and the gut appears to play a significant role in improving ME [7]. Several studies are currently exploring ways to ameliorate ME by modulating the translocation of LPS from the intestines into blood circulation. However, the therapeutic impact of DF on reducing LPS levels in the blood and mitigating low-grade inflammation demonstrates inconsistencies, necessitating further examination of potential underlying factors. Additionally, individual variations in response to dietary interventions and the specific types and amounts of fiber consumed may influence the outcomes. Therefore, our objective is to assess the effect of DF on ME and the severity of low-grade inflammation. By analyzing changes in serum or plasma LPS levels along with inflammatory cytokines, our findings will provide a comprehensive evaluation of available evidence, provide a quantitative summary, identify patterns and trends, enhance statistical power, and address heterogeneity, which collectively will clarify the optimal types, doses, and duration of fiber interventions for managing ME and low-grade inflammation.

## Data Availability

No additional data apart from the supplementary file is available since it is a protocol for a systematic review and meta-analysis.

## Supplementary Materials

The following supporting information can be downloaded at: https://www.mdpi.com/article/10.3390/mps6050084/s1, Table S1: Electronic search strategies.
