# Supplementary material for: Effect of Dietary Fiber Supplementation on Metabolic Endotoxemia: A Protocol for Systematic Review and Meta-Analysis of Randomized Clinical Trials
Source: Methods Protoc. 2023 Sep 11;6(5):84. doi: 10.3390/mps6050084 (PMC10514783; doi:10.3390/mps6050084)
Supplement: Supplementary file 1 [file mps-06-00084-s001.zip › mps-2539493-supplementary.pdf]

Supplementary material

Table S1: Electronic search strategies.

Database	Search Strategy
Cochrane Library	
#1	MeSH descriptor: [Dietary Fibre]
#2	Roughage*
#3	Polysaccharide*
#4	Soluble fibre*
#5	Insoluble fibre*
#6	Prebiotic*
#7	#1 OR #2 OR #3 OR #4 OR #5 OR #6
#8	MeSH descriptor: [Diet]
#9	Diet*
#10	Consume*
#11	Supplementation*
#12	Food*
#13	#8 OR #9 OR #10 OR #11 OR #12
#14	MeSH descriptor: [cellulose]
#15	Lignin*
#16	Hemicellulose*
#17	Hexosane*
#18	#14 OR #15 OR #16 OR #17
#19	Fiber* OR fibre* OR high-fiber* OR high-fibre
#20	Fructans*
#21	Inulin*
#22	Pectin*
#23	MeSH descriptor: [metabolic endotoxemia]
#24	Lipopolysaccharide (LPS)*
#25	Endotoxemia*
#26	Endotoxin-translocation*
#27	Lipopolysaccharide binding protein*
#28	Low-grade inflammation*
#29	Bacterial endotoxin*
#30	Gut permeability*
#31	Toll-like receptor-4*
#32	Systemic inflammation*
#33	#23 OR #24 OR #25 OR #26 OR #27 OR #28 OR #29 OR #30 OR #31 OR #32
#34	#7 AND #33
#35	#13 AND #18 AND #33
Pubmed	
#1	Metabolic endotoxemia OR Endotoxemia
#2	Endotoxemia OR Lipopolysaccharide
#3	Dietary fibre OR dietary Fiber OR Soluble Fibre OR Insoluble Fibre OR Soluble Fiber OR Insoluble Fiber OR Prebiotic OR Inulin OR Polysaccharide

	OR Fructan OR Pectin OR Lignin OR Supplementation
#4	Randomized controlled trial
#5	Controlled clinical trial
#6	Quasi-randomized trial
#7	Cross-over clinical trial
#8	Placebo
#9	Randomly
#10	Groups
#11	Humans
#12	#4 OR #5 OR #6 OR #7 OR #8 OR #9 OR #10
#13	#12 AND #11
#14	#1 AND #2 AND #3 AND #13
MEDLINE OVID	
1.	Exp Dietary fibre/
2.	Roughage*.tw.
3.	Polysaccharide*.tw.
4.	Soluble fibre*.tw.
5.	Insoluble fibre*.tw.
6.	Prebiotic* .tw.
7.	OR /1-6
8.	Diet/
9.	Diet*.tw.
10.	Consume*.tw.
11.	Supplementation*.tw.
12.	Food*.tw.
13.	OR/8-12
14.	Exp cellulose/
15.	Exp Lignin/
16.	Exp Hemicellulose/
17.	Exp Hexosane/
18.	Cellulose* . tw.
19.	Lignin* . tw.
20.	Hemicellulose*.tw.
21.	Hexosane*.tw.
22.	(Fiber* OR fibre* OR high-fiber* OR high-fibre)
23.	Fructans/
24.	Fructans*.tw.
25.	Inulin/
26.	Inulin*.tw.
27.	Pectin/
28.	Pectin*.tw.
29.	OR/14-28
30.	7 AND 13 AND 29
31.	Exp Metabolic Endotoxemia/
32.	Metabolic endotoxemia*.tw.
33.	Endotoxemia*.tw.
34.	Lipopolysaccharide*.tw.

35.	Endotoxin-translocation*.tw.
36.	Lipopolysaccharide binding protein*.tw.
37.	Low-grade inflammation*.tw.
38.	Bacterial endotoxin*.tw.
39.	Gut permeability*.tw.
40.	Toll-like receptor-4*.tw.
41.	Systemic inflammation*.tw.
42.	OR/31-41
43.	30 AND 42
44.	Randomized control trial.pt.
45.	Controlled trial.pt.
46.	Randomized.pt.
47.	Quasi-randomized trial
48.	Cross-over clinical trial
49.	Placebo
50.	Randomly
51.	Groups
52.	Humans
53.	44 OR 45 OR 46 OR 47 OR 48 OR 49 OR 50 OR 51 OR 52
54.	43 AND 53
ClinicalTrials.gov	[Advanced Search]<br>Disease or condition: Metabolic endotoxemia OR endotoxemia OR low-grade inflammation<br>Other terms: dietary fiber OR dietary fibre OR Bran OR Soluble fiber OR Insoluble Fiber OR Roughage ORx fructan OR pectin OR Lignins OR Inulin OR Prebiotic OR Polysaccharide OR supplementation<br>Study type: Clinical Trails (intervention studies)
WHO ICTRP	[Basic search]<br>(Metabolic endotoxemia OR Endotoxemia) AND (dietary fiber OR dietary fibre OR Bran OR Soluble fiber OR Insoluble Fiber OR Roughage ORx fructan OR pectin OR Lignins OR Inulin OR Prebiotic OR Polysaccharide OR supplementation)
